# The diagnostic performance of PET/CT scans for the detection of para-aortic metastatic lymph nodes in patients with cervical cancer: A meta-analysis

**DOI:** 10.1371/journal.pone.0220080

**Published:** 2019-07-18

**Authors:** Weiying Yu, Changgui Kou, Wei Bai, Xiao Yu, Ruixin Duan, Bo Zhu, Yuanyuan Li, Wanqing Hua, Xiaojun Ren, Yanming Yang

**Affiliations:** 1 Department of Epidemiology and Biostatistics, School of Public Health, Jilin University, Changchun, Jilin, China; 2 Department of Radiotherapy, Second Hospital of Jilin University, Changchun, Jilin, China; Karmanos Cancer Institute, UNITED STATES

## Abstract

**Objective:**

We performed a meta-analysis to evaluate the diagnostic value of positron emission tomography/computed tomography (PET/CT) in the detection of para-aortic lymph node metastasis in cervical cancer.

**Methods:**

We searched the PubMed, Embase, Web of Science, Cochrane Library, Chinese Biological Medicine (CBM), Chinese National Knowledge Infrastructure (CNKI), Wanfang and VIP databases in all languages from their inception to September 2018. Stat15.0 software was used to obtain pooled estimates of sensitivity (SEN), specificity (SPE), positive likelihood ratio (PLR), and negative likelihood ratio (NLR) as well as a summary receiver operating characteristic (SROC) curves. Deek‘s funnel plot was used to assess publication bias. QUADAS-2 was used to evaluate the quality of the studies. The protocol for this meta-analysis is registered in PROSPERO (CRD42019115330).

**Results:**

We obtained 14 studies, and the pooled estimates for sensitivity and specificity of PET/CT were 0.71 (95% confidence interval (CI) = 0.54–0.83) and 0.97 (95% CI = 0.93–0.98), respectively. Pooled PLR and NLR were 21.53 and 0.30, respectively. The diagnostic odds ratio (DOR) was70.59, and the area under the curve (AUC) was 0.95.

**Conclusion:**

PET/CT is an effective and important imaging method for the diagnosis of para-aortic lymph node metastasis in early cervical cancer.

## Introduction

Cervical cancer is a major gynecological cancer; it is the fourth most common cancer in women, and the seventh most common cancer overall [1]. At the global level, between 1990 and 2013, the incidence rate of cervical cancer increased to 9% with an estimated 485,000 new cases and 236,000 deaths worldwide in 2013 [2]. In 2015, 526,000 women were diagnosed with cervical cancer worldwide, resulting in 239,000 deaths [3]. Cervical cancer accounts for 7 million Disability-Adjusted Life-Years (DALYs), with 96% coming from years of life lost (YLLs) and 4% from years lived with disability (YLDs).

Although early detection of cervical cancer results in a higher cure rate, this disease is also prone to recurrence [4–6]. Recurrent cervical cancer has a poor prognosis because once the primary treatment fails, the likelihood of a cure for recurrent disease is low [4, 7, 8]. Earlier intervention leads to a better prognosis. The presence of metastasis in pelvic and para-aortic lymph nodes is one of the most important prognostic and high-risk factors for recurrence in cervical cancer [9–13]. In 2018, clinical staging was revised by the International Federation of Gynecology and Obstetrics (FIGO) Committee, and the major change to the current staging system was the incorporation of nodal status into stage III disease staging. This new staging system clearly reflects the importance of lymph node metastasis as a major prognostic factor in cervical cancer [14, 15]. One study suggested that survival rates were significantly lower in patients with nodal metastases than in patients without metastases to lymph nodes, especially para-aortic lymph nodes [16–18], which was reflected by unfavorable overall 3-year survival rates. The three-year survival rate was 94% for patients with negative nodes compared to 64% for patients with positive pelvic nodes and 35% for patients with positive para-aortic nodes [16]. Therefore, detection of the para-aortic lymph node status is essential to ensure appropriate treatment planning and prediction of prognosis for cervical cancer patients.

Another major change in the current staging system is to use imaging and pathological findings, where available, to assign the stage [15]. This also illustrates the necessity of using imaging methods to examine the status of lymph nodes. Currently, positron emission tomography/computed tomography (PET/CT) is widely used in the clinical evaluation of cancers [19–21]. Some studies indicate that PET/CT with 2-[^18^F] fluoro-2-deoxy-d-glucose (^18^F-FDG) exploits the increased utilization of glucose by malignant cells and thereby high uptake of glucose, showing relatively higher diagnostic value.

Previous meta-analyses have investigated the performance of PET/CT in detecting lymph node metastases in cervical cancer patients, but no study has evaluated para-aortic lymph node metastasis. Therefore, we systematically reviewed all available studies in the literature to objectively evaluate the diagnostic value of PET/CT in the detection of para-aortic lymph node metastasis in cervical cancer.

## Materials and methods

### Data sources and keywords

**Fig 1:**
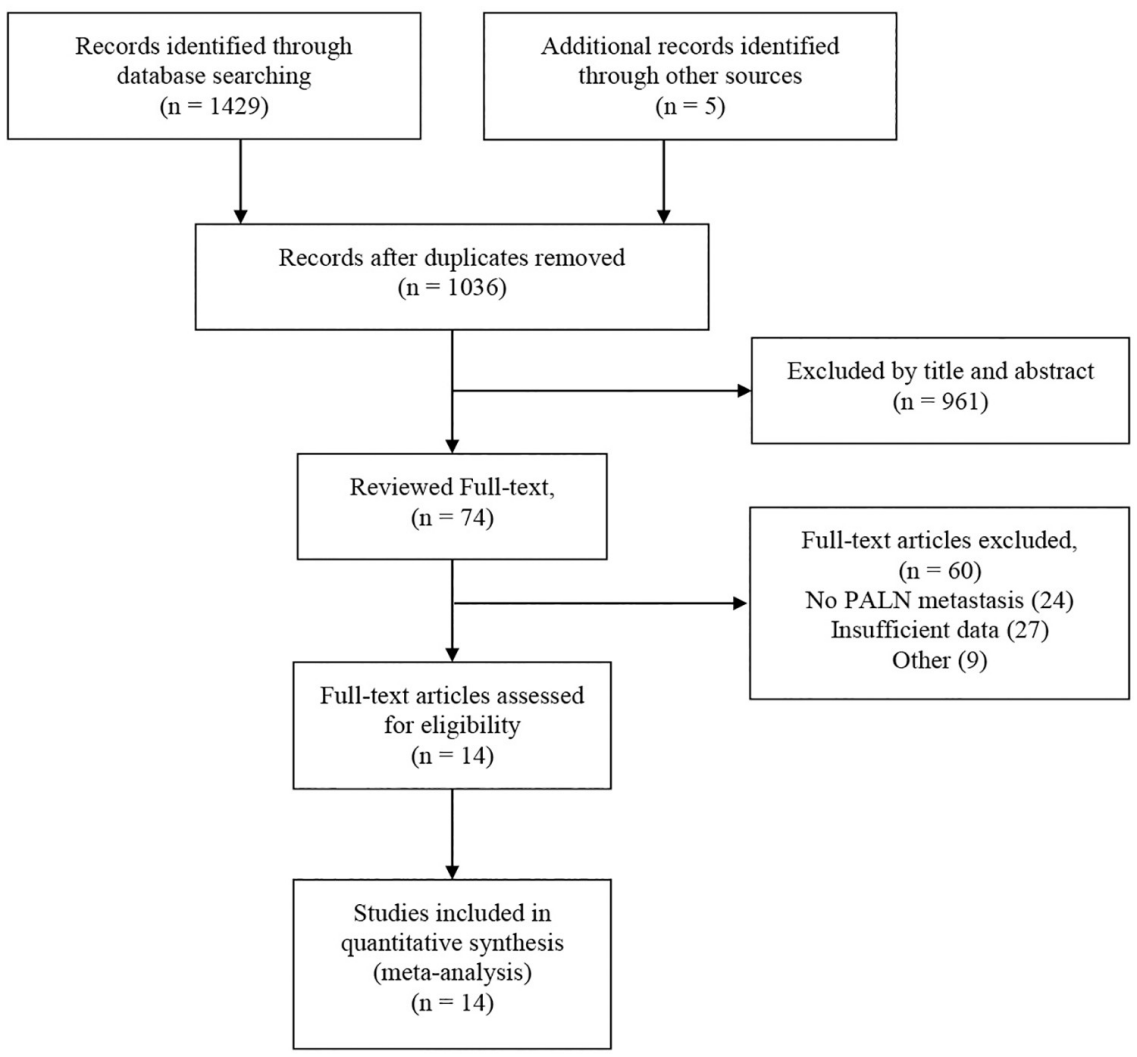
Flow diagram for the selection of studies in this meta-analysis. Abbreviations: PALN, para-aortic lymph nodes.

We searched PubMed, Embase, Web of Science, Cochrane Library, Chinese Biological Medicine (CBM), Chinese National Knowledge Infrastructure (CNKI), Wanfang and VIP databases in all languages from their inception to September 2018. We used a combination of medical subject headings (MeSH) terms and keyword searches. We used the latter to identify relevant papers where the MeSH terms had not been assigned. For the MeSH search, we specified the terms “Uterine Cervical Neoplasms [MeSH]” AND “Positron Emission Tomography Computed Tomography [MeSH]” AND “Lymphatic Metastasis [MeSH]”. These terms were expanded in the search builder to “Uterine Cervical Neoplasms” OR “Cervical Neoplasm, Uterine” OR “Cervical Neoplasms, Uterine” OR “Neoplasm, Uterine Cervical” OR “Neoplasms, Uterine Cervical” OR “Uterine Cervical Neoplasm” OR “Neoplasms, Cervical” OR “Cervical Neoplasms” OR “Cervical Neoplasm” OR “Neoplasm, Cervical” OR “Neoplasms, Cervix” OR “Cervix Neoplasms” OR “Cervix Neoplasm” OR “Neoplasm, Cervix” OR “Cancer of the Uterine Cervix” OR “Cancer of the Cervix” OR “Cervical Cancer” OR “Uterine Cervical Cancer” OR “Cancer, Uterine Cervical” OR “Cancers, Uterine Cervical” OR “Cervical Cancer, Uterine” OR “Cervical Cancers, Uterine” OR “Uterine Cervical Cancers” OR “Cancer of Cervix” OR “Cervix Cancer” OR “Cancer, Cervix” OR “Cancers, Cervix” AND “Positron Emission Tomography Computed Tomography” OR “PET-CT Scan” OR “PET-CT Scans” OR “Scan, PET-CT” OR “Scans, PET-CT” OR “PET CT Scan” OR “CT Scan, PET” OR “CT Scans, PET” OR “PET CT Scans” OR “Scan, PET CT” OR “Scans, PET CT” OR “CT PET” OR “CT PETs” OR “PET, CT” OR “PETs, CT” OR “Positron Emission Tomography-Computed Tomography” OR “PET-CT” OR “CT PET Scan” OR “CT PET Scans” OR “PET Scan, CT” OR “PET Scans, CT” OR “Scan, CT PET” OR “Scans, CT PET” AND “lymph node metastases” OR “Lymphatic Metastasis” OR “Lymphatic Metastases” OR “Metastases, Lymphatic” OR “Metastasis, Lymphatic”. Meanwhile, references from the retrieved papers were checked for additional relevant studies. Potentially relevant articles were identified based on the title and abstract and predefined inclusion and exclusion criteria. The flow diagram of the retrieval process is shown in Fig 1. The protocol for this meta-analysis is registered in PROSPERO (CRD42019115330).

### Inclusion and exclusion criteria

Studies were eligible according to the following criteria: (1) A sample size was at least 10 patients. (2) PET/CT or PET was used to detect para-aortic lymph node metastasis in patients with cervical cancer. (3) Histopathologic findings were the “gold standard”. (4) The provided data included true positives (TP), false positives (FP), false negatives (FN) and true negatives (TN).

Studies were excluded according to the following criteria: (1) Reviews, letters, case reports, nonclinical studies; (2) Insufficient data (2×2 tables could not be constructed); (3) Para-aortic lymph node metastasis was not confirmed by pathology; and (4) Studies with duplicate data.

### Data extraction

Two authors independently extracted the data from each relevant article. Any disagreement between the authors over the eligibility of particular studies was resolved through discussion with a third reviewer. For each included study, we recorded the name of the first author, year of publication, number of eligible patients, comparison methods (patient-based and region-based), true positive (TP), false positive (FP), false negative (FN), true negative (TN) and information for the assessment of the risk of bias. Missing data were requested from the study authors.

### Statistical analyses

Stata 15.0 was utilized to analyze the data. A bivariate random effects model was performed to adjust for the within and between-study variance in sensitivity and specificity. We extracted the data from diagnostic 2×2 tables in the study to calculate pooled estimates of sensitivity(SEN),specificity(SPE), positive-likelihood ratios (PLR), negative-likelihood ratios (NLR), and the diagnostic odds ratio (DOR) with 95% confidence interval (CI). We plotted a summary receiver operating characteristic (SROC) curves, obtained the area under the curve (AUC), and constructed a forestplot. The diagnostic value was better when the AUC was closer to 1. We assessed the threshold effect using Spearman’s correlation coefficients and evaluated heterogeneity based on the *I*-square (*I*^*2*^) values. The possibility of publication bias was assessed by a Deek’s funnel plot[22], a symmetric shape of the funnel plot indicates that there is no publication bias, because visual inspection is subjective, and it is necessary to quantify the evidence for asymmetry. *P*>0.05 was considered to be indicative of no publication bias.

### Quality assessment

Two reviewers independently assessed the risk of bias and the applicability of diagnostic accuracy in the studies using the QUADAS-2 method [23] which consists of four main domains: (1) patient selection, (2) index test, (3) reference test, and (4) flow and timing. Each domain was assessed in terms of the risk of bias (high, low or unclear), and the first 3 domains were also assessed in terms of concerns regarding applicability. Disagreements were resolved by discussion with a third review when necessary.

## Results

### Eligible studies and quality assessment

**Fig 2:**
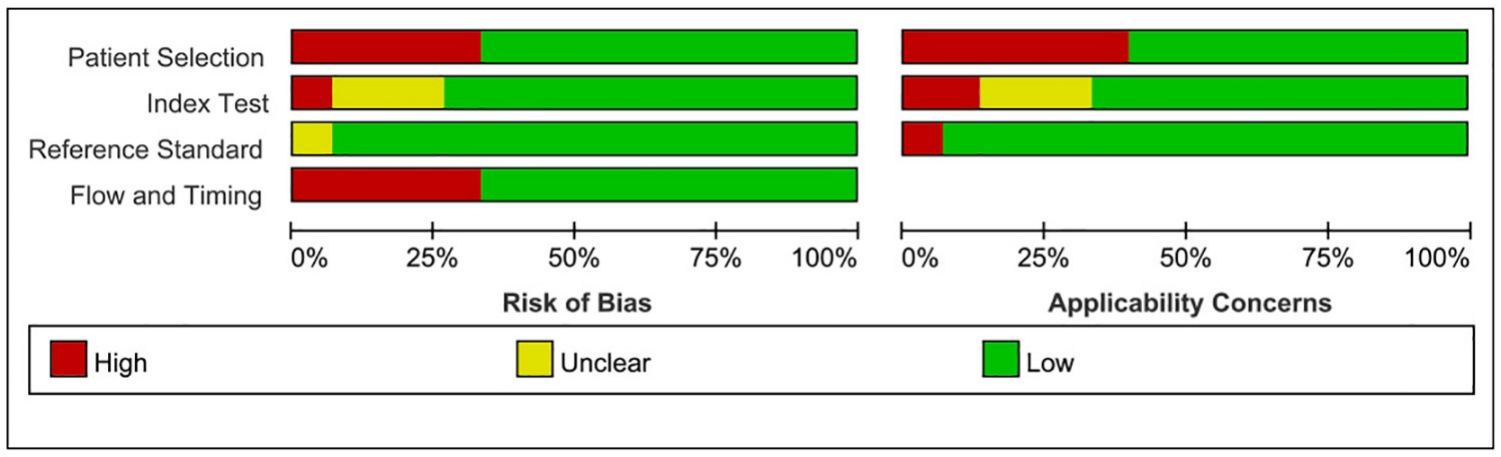
Methodological quality of the studies included in this meta-analysis.

The initial search yielded 1429 studies (PubMed: 430; Embase: 329; Web of Science: 587; Cochrane Library: 7; Chinese Biological Medicine (CBM): 13; Chinese National Knowledge Infrastructure (CNKI): 39; Wanfang: 9; VIP: 15), and another 5 articles were found by manual searching. A total of 398 articles were excluded based on duplication, and 962 studies were excluded based on the title or abstract. After that, we reviewed the remaining 74 full-text articles. Finally, 14 studies remained and were eligible for this meta-analysis. The flow diagram of the retrieval process is shown in Fig 1, and the characteristics of the 14 studies are shown in Table 1. We assessed the quality of the 14 articles according to the QUADAS-2 assessment tool (Fig 2).

**Table 1:** The general characteristics of the included studies.

Studies	Sample	TP	FP	FN	TN	SEN (%)	SPE (%)	PPV (%)	NPV (%)
Rose1999[24]	32	6	2	2	22	75.00	91.67	75.00	91.67
Narayan2001[25]	24	4	1	3	16	57.14	94.12	80.00	84.21
Yeh2002[26]	42	10	1	2	29	83.33	96.67	90.91	93.55
Lin2003[27]	50	12	2	2	34	85.71	94.44	85.71	94.44
Ryu2003[28]	249	3	0	1	245	75.00	100.00	100.00	99.59
Wright2005[29]	45	1	1	3	40	25.00	97.56	50.00	93.02
Loft2007[30]	119	15	1	0	103	100.00	99.04	93.75	100.00
Kitajima2008[31]	52	10	0	2	40	83.33	100.00	100.00	95.24
Yildirim2008[32]	16	2	2	2	10	50.00	83.33	50.00	83.33
Monteil2011[33]	27	5	5	0	17	100.00	77.27	50.00	100.00
Leblanc2011[34]	125	7	6	14	98	33.33	94.23	53.85	87.50
Ramirez2011[35]	60	5	2	9	44	35.71	95.65	71.43	83.02
Chen2013[36]	45	2	1	3	39	40.00	97.50	66.67	92.86
Ge2015[37]	26	7	1	2	16	77.78	94.12	87.50	88.89

Abbreviations:TP,true positive; FP,false positive; FN,false negative; TN,true negative; SEN,sensitivity; SPE,specificity; PPV,positive predictive value; NPV,negative predictive value

### Effect of PET/CT on diagnosis

**Fig 3:**
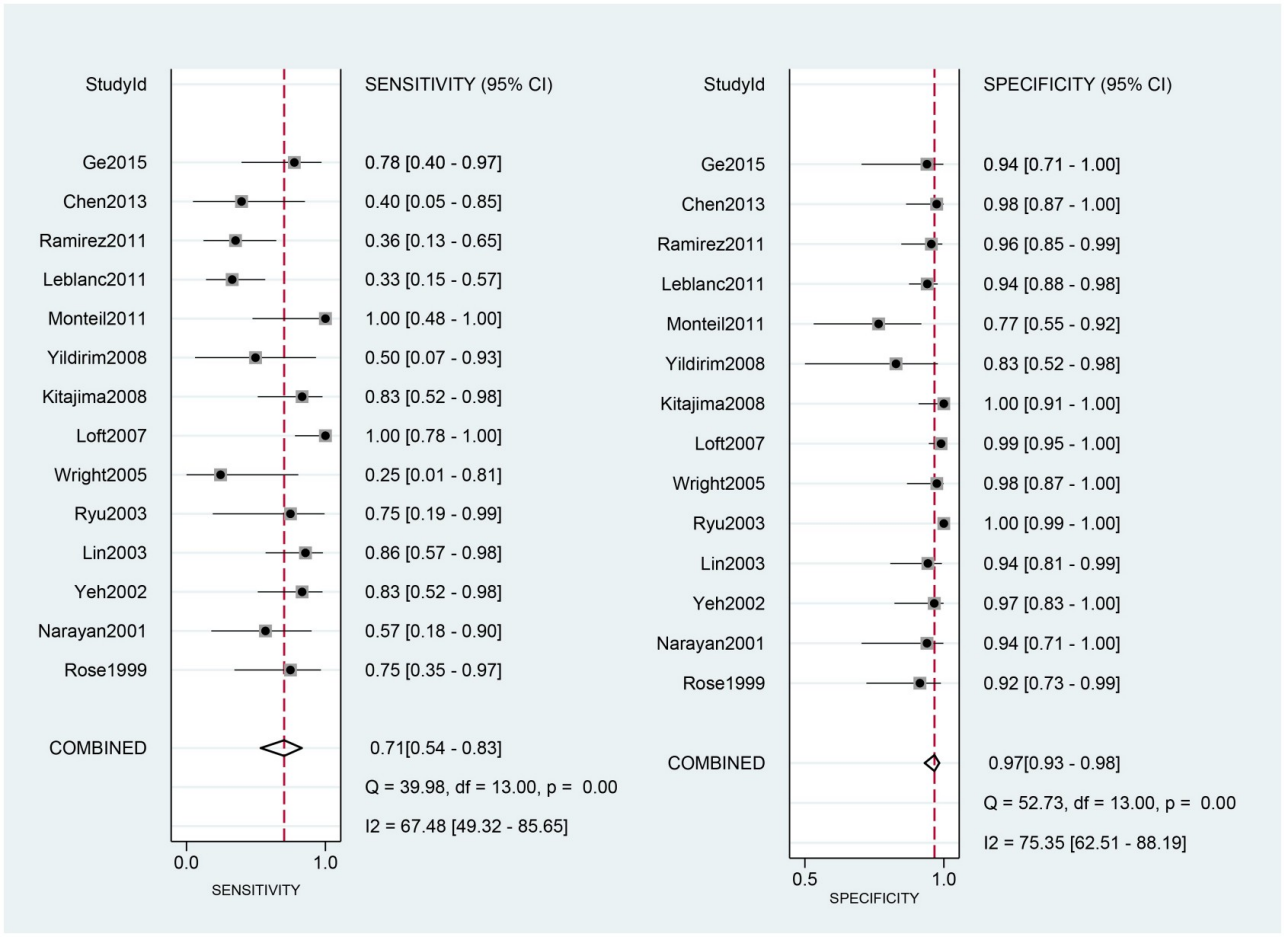
Forest plots for sensitivity and specificity. Abbreviations: CI, confidence interval; df, degrees of freedom.

**Fig 4:**
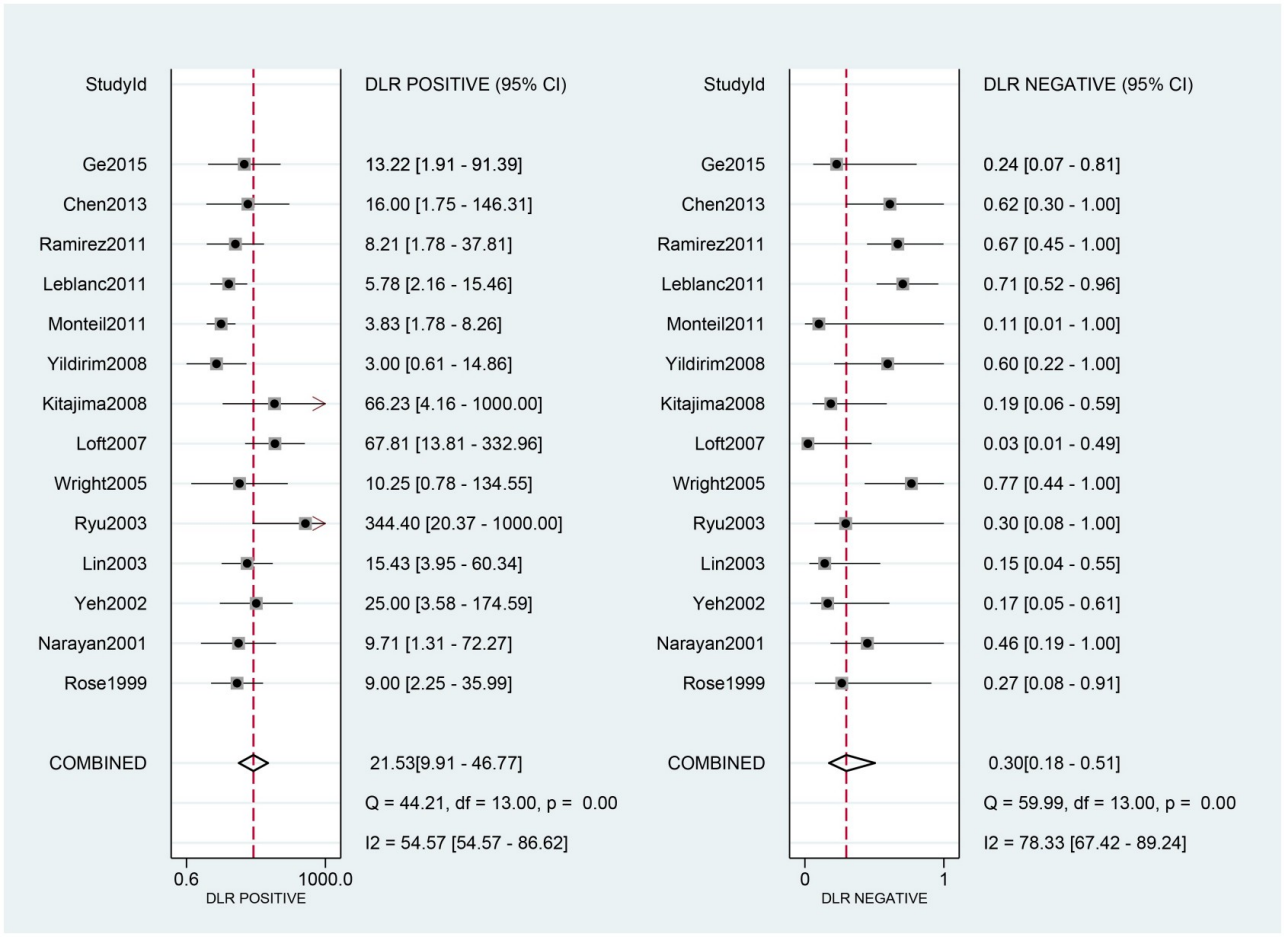
Forest plots for positive and negative LRs. Abbreviations: CI, confidence interval; df, degrees of freedom; LR, likelihood ratio.

The 14 eligible studies were pooled for the meta-analysis of diagnostic test. As moderate heterogeneity among the 14 studies was observed (*I*^2^ = 68, 95% CI: 28–100). Fig 3 shows the forest plots of sensitivity, ranging from 0.25 to 1.00 (pooled 0.71; 95% CI: 0.54–0.83), and specificity, ranging from 0.77 to 1.00 (pooled 0.97; 95% CI: 0.93–0.98). The pooled positive-likelihood ratio (PLR) was 21.53, and the pooled negative-likelihood ratio (NLR) was0.30 (Fig 4).

**Fig 5:**
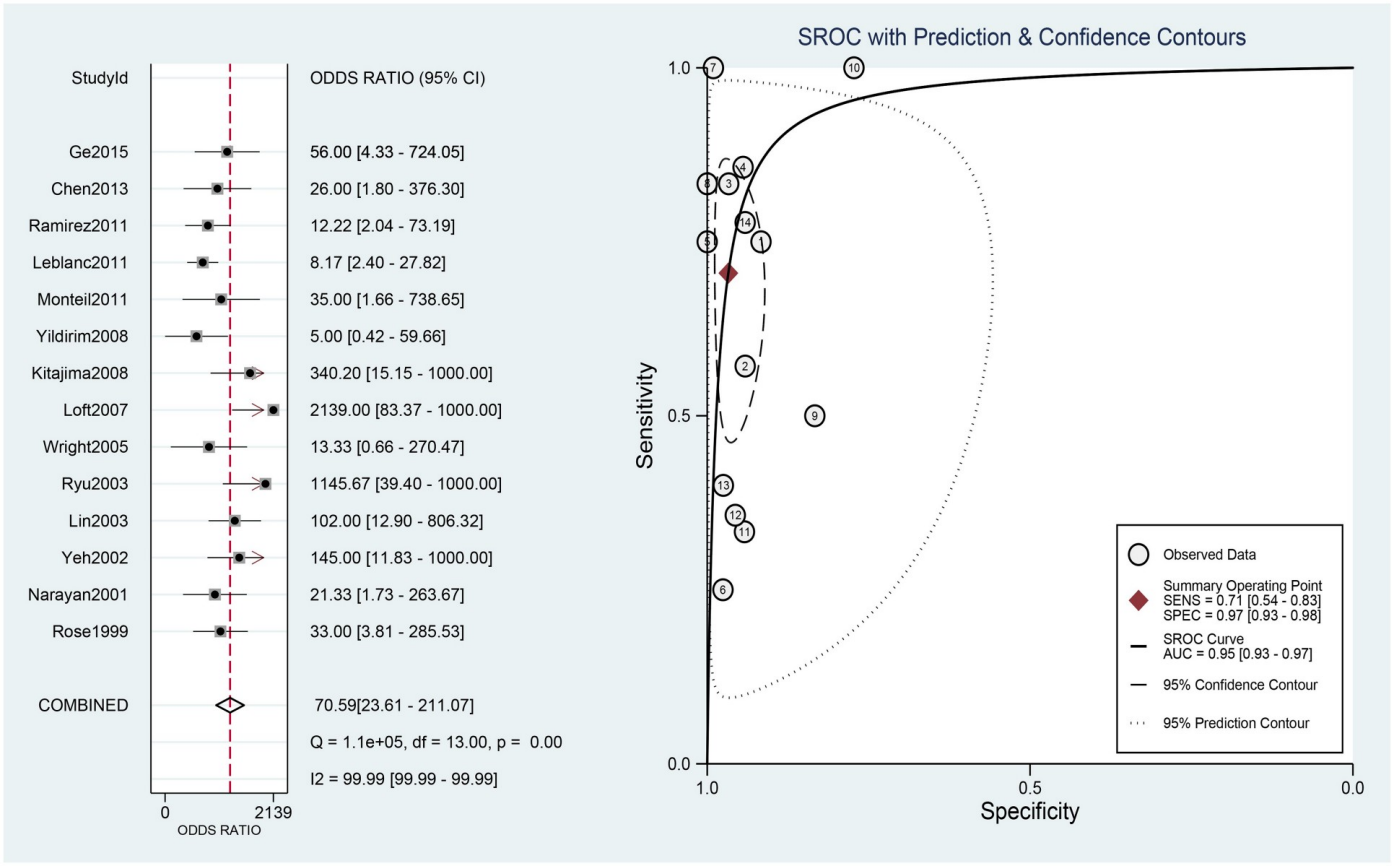
Forest plots for the DOR and the SROC curve. Abbreviations: CI, confidence interval; df, degrees of freedom; DOR, diagnostic odds ratio; AUC, area under the curve; SROC, summary receiver operating characteristic.

Fig 5 shows the diagnostic odds ratio (DOR) ranging from 5.00 to 2139.00 (pooled 70.59; 95% CI: 23.61–211.07). The area under the curve (AUC) for PET/CT was 0.95 (0.93–0.97).

### Publication bias

**Fig 6:**
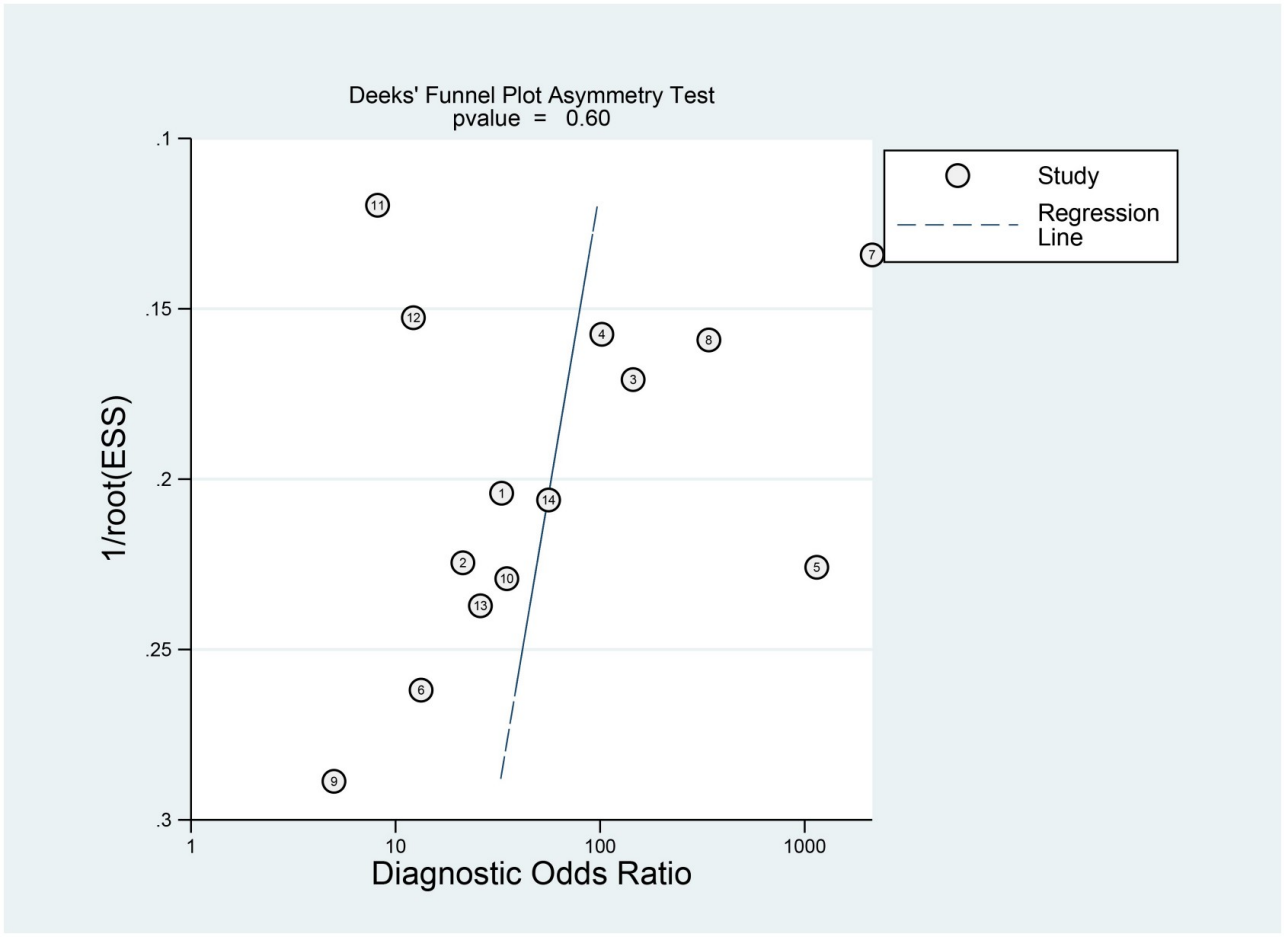
Funnel plot of the diagnostic value of PET/CT.

Publication bias was evaluated by a Deek’s funnel plot (Fig 6), reflected by the symmetric shape of the funnel plot. The P value was 0.60, which indicated a low probability of publication bias.

## Discussion

Cervical cancer is the most commonly diagnosed cancer for women in 11 countries, and the most common cause of cancer deaths for women in 50 countries [3]. Para-aortic lymph node metastases are associated with an unfavorable prognosis in cervical cancer patients. In the new FIGO clinical staging guidelines, lymph node status is included [15]. Para-aortic lymph node metastasis must be detected accurately to plan appropriate treatment for cervical cancer patients.

In this meta-analysis, a total of 14 original studies were included. Since each study has a limited number of cases, this meta-analysis was performed to integrate more data and provide more reliable results. Some meta-analyses have been published on lymph node metastasis in cervical cancer, but our meta-analysis is the first to evaluate the diagnostic performance of PET/CT for detecting para-aortic lymph node metastases in cervical cancer patients. We performed a meta-analysis and strictly followed the inclusion and exclusion criteria to guarantee the quality of all included studies. We also examined the methodological quality of the studies using the validated QUADAS-2 tool.

Histopathologic findings are the “gold standard” to determine the lymph node status; however, the benefits of lymphadenectomy are controversial. In this scenario, different imaging modalities have been used to study the presence of lymph node metastasis by a noninvasive approach. Various imaging methods such as computed tomography (CT) [38] and magnetic resonance imaging (MRI) [39, 40] have traditionally been used to detect the lymph node status in cervical cancer. Nevertheless, the use of CT and MRI techniques to detect metastasis to lymph nodes remains difficult and it reflected in the low sensitivity values of CT and MRI in the detection of nodal metastasis [38, 41]. Some studies have also evaluated the role of PET/CT in the clinical evaluation of cancers. PET/CT has been indicated to be superior to conventional imaging modalities in the detection of lymph node metastasis [42].

We investigated the diagnostic performance of PET/CT for the detection of para-aortic lymph node metastases in cervical cancer patients with surgical pathology as a reference standard. A previous meta-analysis showed that PET/CT had moderate sensitivity and good specificity in detecting lymph node metastasis of cancer [43–45]. In a study by Atri et al [46], PET/CT was demonstrated to have a good effect on the diagnosis of abdominal lymph node metastasis in patients with cervical cancer, and the ACRIN6671/GOG0233 trial was one of the reasons for moving toward the adoption of the new FIGO staging system, which clearly proposes the use of imaging and pathological findings, where available, to assign the stage [15]. The pooled estimates for sensitivity and specificity of PET/CT were 0.71 (95% CI = 0.54–0.83) and 0.97 (95% CI = 0.93–0.98), respectively. Thus, PET/CT has moderate sensitivity and high specificity, but the rate of missed diagnosis is 29% and the misdiagnosis rate is 3%. We found similar SPE and SEN to the meta-analysis of PET/CT for lymph node metastasis in cervical cancer patients performed by Ruan et al [47], but we found higher SEN and lower SPE than the values obtained in their subgroup analysis of para-aortic lymph node metastasis. The overall results in the study by Liu et al [48] are similar to those in the subgroup analysis, and have lower SEN that and similar SPE to our results. The difference in overall results may be due to the inclusion of pelvic lymph node metastasis in the other studies. Although they performedsubgroup analyses, they included fewer studies of para-aortic lymph node metastasis, which may have accounted for the differences in results.

The DOR in this study 70.59. DOR has the advantage of accuracy as a single indicator and combines the strengths of SEN and SPE. The DOR ranges from 0 to infinity, with higher values indicating better discriminatory performance [49–51]; however, the DOR is difficult to apply directly to clinical practice [52]. Since likelihood ratios (LR) are considered more clinically meaningful, we also calculated both PLR and NLR and used them as measures of diagnostic accuracy. In this meta-analysis, the pooled PLR and NLR were 21.53 and 0.30, respectively. Positive and negative likelihood ratios describe the discriminatory properties of positive and negative test results, respectively. Positive likelihood ratios above 10 and negative likelihood ratios below 0.1 have been noted to provide convincing diagnostic evidence [51]. In this study, the positive likelihood ratio was greater than 10, and could be used for effective diagnosis, but the negative likelihood ratio was not lower than 0.1, and could not be used to directly exclude the disease. The area under the SROC curve was 0.95, and the value was close to 1. These data showed that PET/CT can be used as an effective diagnostic method to detect para-aortic lymph node metastasis in cervical cancer patients. The *I*^2^ value was 68(CI = 20–100), indicating moderate heterogeneity. Nevertheless, because of incomplete data, we could not perform subgroup analyses, and we could not present the exact reasons for heterogeneity.

This meta-analysis had several limitations. First, not all studies in this meta-analysis reported the specific technology utilized. Some scanning parameters may have affected the accuracy of PET/CT. Second, most of the studies were retrospective and did not use a blinded method to obtain results, which may have influenced the quality of the studies. Third, although a low probability of publication bias, the number of articles included was small, and the lack of studies from recent years may have caused bias. Last, the number of patients included in the studies was relatively small, which might have caused bias in the final results.

In summary, despite these limitations, PET/CT showed good diagnostic performance for the detection of para-aortic lymph node metastasis in patients with cervical cancer. As an effective and important imaging method for the discovery of para-aortic lymph node metastasis to make an early diagnosis of cervical cancer, PET/CT can be used to ensure appropriate treatment planning and preoperative assessment.

## Conclusion

Our meta-analysis shows that PET/CT is an effective and important imaging method for the diagnosis of para-aortic lymph node metastasis in early cervical cancer. Therefore, the application of PET/CT in the clinic will be beneficial for the treatment and prognostic assessment of cervical cancer patients.

## Supporting information

S1 File PRISMA checklist. (DOCX)
